# Hypodermic Injections of Morphia

**Published:** 1863-02

**Authors:** 


*Hypodermic Injections of Morphia.*—Dr. Lerick, of Philadelphia, reports to the Phil. Coll. of Phys., the use of Hypodermic injections of Magendie's solution of morphia (gr. xvj to f ℥ j) in a case of excessively obstinate neuralgia of the thigh and leg, supervening on myelitis. Fifteen, gradually increased to twenty-eight, minims were injected each time; sometimes only at night, sometimes twice or three times a day. The treatment was continued for five months. All other treatment was gradually abandoned as useless. In five minutes the patient, who was previously in an agony of suffering, would be perfectly relieved. No unpleasant sequelæ resulted. He does not hesitate to recommend the method as a very valuable addition to our means of medication. Similar injections have been recommended in delirium tremens, tetanus, mania, chorea, paralysis, &c., and they illustrate the fact that the anodynes have a powerful influence in controlling nervous disorder, even thus irregularly applied. We trust there will be no indiscriminate resort to them. Occasionally abscesses are the result. Sometimes, not infrequently, nausea and vomiting. The narcotic effect is not readily controlled, now and then there occurs profound impression, and again from even a larger amount very little or none.

Still in obstinate cases this method of application is worthy trial. Where temporary or permanent causes prevent ordinary methods of exhibition, or it is sought to impress at every point. So in metastatic pain; in surgical cases; but especially where pain is distinctly local and chronic. Practically it is best to inject directly upon the most painful point, although some, from theoretical considerations, prefer to apply the medicine at the trunk of the nerve whose branches are specially involved. By the by, an appropriate instrument has yet to be invented for this little operation. Those we have yet seen are often imperfect at the first, and always after having been used a few times. The little ones, guaged for sundry minims, in a short time infallibly draw air from above, rather than the narcotic solution through the fine tube from below. All the vulcanized rubber syringes, although the tube remains intact, speedily give way at the soft packing of the piston. Can not this be remedied?

It is a little noteworthy that in about all the cases reported where hypodermic injections were resorted to, almost as the last hope of relief, the efforts of the physician have been directed to finding some specific remedy, apparently guided by the idea of *functional* (*quasi* spiritual) disease. Little is said of efforts to restore a healthy composition of the blood. Good healthy blood is the best healing lotion for even "functional" diseases.

